# A Practical Treatise on the Management and Diseases of Children

**Published:** 1843-07-08

**Authors:** 


*A Practical Treatise on the Management and Diseases of Children.* By Richard G. Evanson, M. D., Professor of Medicine in the Royal College of Surgeons, Ireland, and Henry Maunsell, M. D., Professor of Political Medicine in the Royal College of Surgeons, Ireland. Second American, from the fourth Dublin Edition, with Notes, by D. Francis Condie, M. D., Fellow of the College of Physicians of Philadelphia, &c. Philadelphia: Ed. Barrington & George D. Haswell. 1843. 8vo. pp. 372.

This publication is familiar to the American Medical public, through a former edition. The present edition is enlarged, and in its present form constitutes one of the best works on the subject in our language. We do not intend to say that it is by any means unexceptionable, but that at this time we know of no English production on the same subject which is superior, or perhaps, on the whole, as good. Dr. Condie has added a few notes, together with an excellent chapter on Gangrene of the Mouth in Children, and he has judiciously extended a section which treats of Cholera Infantum, the description given by the authors showing that they were treating of a disease differing widely from the formidable affection known in this country. Dr. Condie might, we think, have been less sparing of his emendations and additions, and by so doing he would have supplied several lamentable deficiencies. The chapter on Pneumonia is very imperfect, and the important, and by no means uncommon affection of Tubercular Meningitis is passed over in the following manner: "Dr. Gerhard describes tubercular deposition on the membranes. We have certainly seen some cases of this description." p. 287. Both diseases are, we suspect, by no means uncommon in Ireland, and we are surprised that the omission of the authors has not been supplied by the editor.

On the whole the work is a very excellent one; indeed no better manual is at present within reach of the student and practitioner.

